# Copy number variation genotyping using family information

**DOI:** 10.1186/1471-2105-14-157

**Published:** 2013-05-09

**Authors:** Jen-hwa Chu, Angela Rogers, Iuliana Ionita-Laza, Katayoon Darvishi, Ryan E Mills, Charles Lee, Benjamin A Raby

**Affiliations:** 1 Channing Division of Network Medicine, Brigham and Women’s Hospital, Boston MA, USA; 2 Department of Pathology, Molecular Genetic Research Unit, Brigham and Women’s Hospital, Boston MA, USA; 3 Division of Pulmonary and Critical Care Medicine, Brigham and Women’s Hospital, Boston MA, USA; 4 Center for Genomic Medicine, Brigham and Women’s Hospital, Boston MA, USA; 5 Mailman School of Public Health, , NY, New York, USA; 6 Department of Human Genetics, University of Michigan, MI, Ann Arbor, USA

## Abstract

**Background:**

In recent years there has been a growing interest in the role of copy number variations (CNV) in genetic diseases. Though there has been rapid development of technologies and statistical methods devoted to detection in CNVs from array data, the inherent challenges in data quality associated with most hybridization techniques remains a challenging problem in CNV association studies.

**Results:**

To help address these data quality issues in the context of family-based association studies, we introduce a statistical framework for the intensity-based array data that takes into account the family information for copy-number assignment. The method is an adaptation of traditional methods for modeling SNP genotype data that assume Gaussian mixture model, whereby CNV calling is performed for all family members simultaneously and leveraging within family-data to reduce CNV calls that are incompatible with Mendelian inheritance while still allowing de-novo CNVs. Applying this method to simulation studies and a genome-wide association study in asthma, we find that our approach significantly improves CNV calls accuracy, and reduces the Mendelian inconsistency rates and false positive genotype calls. The results were validated using qPCR experiments.

**Conclusions:**

In conclusion, we have demonstrated that the use of family information can improve the quality of CNV calling and hopefully give more powerful association test of CNVs.

## Background

Copy Number Variants (CNV) are DNA segments whose copy-number deviates from the expected two copies observed in diploid genomes [1,2]. CNVs represent the most common form of structural genetic variation and their importance in genetic disease has been established [3]. A large number of common polymorphic CNVs that segregate at fixed frequencies in human populations have been discovered, several of which have demonstrated reproducible associations with complex genetic diseases, including susceptibility to autoimmune and neuropsychiatric diseases, cancer and asthma [2,4-7]. There is therefore great interest in developing high-throughput CNV genotyping arrays and statistical methods to enable genome-wide screens for CNV association with disease.

Technologies have been developed for both CNV discovery and genotyping, the majority of which are array based, including comparative genomic hybridization (CGH) or SNP genotyping arrays [8]. In contrast to CNV discovery arrays, association (i.e. genotyping) arrays are designed to target specific genome segments known to harbor previously identified CNVs, often with substantially fewer probes spaced at much lower density. As such, standard statistical methods for CNV discovery arrays, such as segmentation based methods [9] and Hidden Markov models (HMM) [10-12], may not be appropriate for CNV association arrays, as these models rely on the associations between closely-spaced adjacent probes. For CNV association assays, Barnes et al [13] developed a statistical framework for CNV calling in case-control association studies, which has been applied in a large-scale genome wide association study of 8 diseases, the Wellcome Trust Case Control Consortium (WTCCC) study [14]. However, the WTCCC study did not identify any disease-relevant CNVs that had not been previously identified in SNP-based studies. Evidently one of the most challenging problems in CNV studies is the data quality. The CGH array used for the WTCCC study contained 105,000 probes targeting 12,000 CNV regions, and after thorough QC filtering only 3,432 regions were considered for association analysis, as the majority of the regions were either not variable or could not be called with sufficient confidence. The data quality problem is not limited to CGH array but an issue for SNP genotyping array as well [15,16].

One way to help overcome such data quality issue is to use the family-based design for genetic associations. When available, family data can be incorporated to improve copy-number assignment of genotyped CNVs. In this paper we introduce a statistical framework for family-based CNV studies based on the Gaussian mixture model described in [13,17]. Our method assigns copy-number for all members of a nuclear family simultaneously, leveraging the familial relationships to reduce copy-number calls that are incompatible with Mendelian inheritance while still allowing for the presence of occasional de-novo CNVs. We demonstrate our method with an application to a CNV genome-wide association study in asthma. Using experimentally validated data, we found that our method not only can significantly reduce the Mendelian inconsistency, but also improve the copy-number assignment accuracy compared to existing methods. This extra step of “data cleaning” can be crucial to the downstream association tests [18,19].

## Methods

### Gaussian mixture model

We model the log2 ratios distribution with the Gaussian mixture model (GMM) described in [20] and [13]. We assume that the data (log2 ratios) {*y*_1_,..,*y*_*n*_} are generated from a mixture model with *G* components

$$f(y)=\overset{n}{\underset{i=1}{\prod }}\overset{G}{\underset{k=1}{\sum }}\tau_{k}f_{k}(y_{i}|\theta_{k}),$$

where *f*_*k*_(*y*_*i*_|*θ*_*k*_) is normal distributions with mean *μ*_*k*_ and variance $\sigma_{k}^{2}$

$$f_{k}(y_{i}|\theta_{k})={\left(2\Pi \sigma_{k}^{2}\right)}^{1/2}\;\exp\;\left\{-\frac{{\left(y_{i}-\mu_{k}\right)}^{2}}{2\overset{2}{\underset{k}{\sigma }}}\right\},$$

with *θ*_*k*_=(*μ*_*k*_,*σ*_*k*_). The components 1,..G correspond to discrete copy numbers (0,1,2...). The parameters of the model {*τ*_*k*_,*μ*_*k*_,*σ*_*k*_} can be estimated using the E-M (Expectation-Maximization) algorithm, described in [17,20]. The E-M is a general approach to maximum likelihood estimation for missing data problems. In our case the “missing data” is the unobserved assignment of clusters for the samples *z*_*ik*_:

$$z_{\text{ik}}=\left\{\begin{array}{l} 1\;\text{if sample}\;i\;\text{belongs to cluster}\;k\\ 0\;\text{otherwise}. \end{array}\right)$$

Then the “complete data” log likelihood becomes:

$$L(\theta ,\tau |z,y)=\overset{n}{\underset{i=1}{\sum }}\overset{G}{\underset{k=1}{\sum }}z_{\text{ik}}\;\log\;\{\tau_{k}f_{k}(y_{i}|\theta_{k})\}.$$

The E-step (Expectation): Computing the conditional probability of sample *i* belongs to cluster *k*

$${\hat{z}}_{\text{ik}}=\frac{\tau_{k}f_{k}(y_{i}|\theta_{k})}{\sum_{j=1}^{G}\tau_{j}f_{j}(y_{i}|\theta_{j}).}$$

The M-step (Maximization): The parameters are estimated given the conditional probability *z*_*ik*_.

$${\hat{\mu }}_{k}={\bar{y}}_{k},{\hat{\sigma_{k}}}^{2}=\frac{\sum_{j=1}^{n}z_{\text{jk}}{\left(y_{i}-{\bar{y}}_{k}\right)}^{2}}{n_{k}},\hat{\tau_{k}}=\frac{n_{k}}{n},$$

with $n_{k}=\sum_{j=1}^{n}z_{\text{jk}}$ and ${\bar{y}}_{k}=\overset{n}{\underset{j=1}{\sum }}(z_{\text{jk}}y_{j}/n_{k})$.

The E-step and M-step are iterated until convergence.

We use the R package mclust [17] for implementation of the E-M algorithm. We fit each region level summary with up to 5 clusters and assign each cluster with discrete copy numbers, with the largest cluster assumed to be the normal (two-copy) group in most cases. The clusters below 0 copy and above 4 copies are merged into adjacent groups.

### Incorporating family data

To appropriately model the probabilities of specific parent-child copy-number configurations, we use the following probabilistic model from [21] and introduce two additional parameters: *a* is the probability of the rare chromosome-specific copy number configuration (See Table 1), and *e* is the probability of de-novo mutation. Both probabilities should be small, but greater than zero, to support all possible configurations of copy numbers in a trio. In other words, any combination of copy numbers from 0 to 4 copies will have a non-zero probability a priori, even though some probabilities will be very small. The CNV inheritance matrix, i.e. the conditional distribution of the children’s copy numbers given the parents’, can be specified with these two parameters (Additional file 1: Table S1).

**Table 1:** **Probabilistic model specifying chromosome-specific copy number at a single marker given the total copy number**[21]

**Total** **copy number**	**Chromosome-specific** **copy number**	**Probability**
0	0/0	1
1	0/1	1
2	1/1(common form)	1−*a*
	0/2 (rare form)	*a*
3	1/2 (common form)	1−*a*
	0/3 (rare form)	*a*
4	2/2	0.5
	1/3	0.5

Let *z*^*f*^,*z*^*m*^ and *z*^*o*^ represent the copy number distribution for the father, mother and offspring, respectively. The posterior probability of the trio

$$\begin{array}{lll} P(z^{o},z^{f},z^{m}|y,\tau ,\theta ) & =\underset{g\in \{o,f,m\}}{\prod }p(y^{g}|z^{g},\tau ,\theta )P(z^{o}|z^{f},z^{m})\; & \;\\ & \;\;\;\;p(z^{f}|\tau ,\theta )p(z^{m}|\tau ,\theta )\; & \; \end{array}$$

where *P*(*z*^*o*^|*z*^*f*^,*z*^*m*^) is the inheritance probability in the CNV inheritance matrix. Therefore, in the E-M algorithm we can simply reweight the E-step for the offsprings:

$$({\hat{z}}_{\text{ik}}^{o}|z_{\text{ik}}^{f},z_{\text{ik}}^{m})=\frac{\tau_{k}P(z_{\text{ik}}^{o}|z_{\text{ik}}^{f},z_{\text{ik}}^{m})f_{k}(y_{i}|\theta_{k})}{\sum_{j=1}^{G}\tau_{j}P(z_{\text{ij}}^{o}|z_{\text{ij}}^{f},z_{\text{ij}}^{m})f_{j}(y_{i}|\theta_{j}).}$$

to obtain the conditional probability distribution of the offspring. The parents’ probability distribution will not be affected in this step. When we perform the M-step the joint conditional probability of the trio *P*(*z*^*o*^,*z*^*f*^,*z*^*m*^|*y*,*τ*,*θ*) is maximized. Therefore it should converge to a model that is more consistent with Mendelian inheritance, but still allowing errors and de-novo events.

### Applied dataset

The study population has been described previously [22-24]. In total, 1211 subjects, including 385 asthmatic children of self-described white ethnicity and their available parents, were genotyped using a custom-designed Agilent 180k probe CGH array for a genome-wide CNV association study of asthma. Regions were selected based on data on CNV location and breakpoints from multiple datasets, in a tiered approach, favoring high-resolution data. We incorporated CNV regions identified by the Structural Genomic Variation Consortium based on data from 42 million CGH probes [25], data from the June 2009 release of the 1000 genomes project [26], deep sequencing of an individual genome [27] and a list of segmental duplications [28] and novel insertions [29]. Finally, we incorporated variants identified in the Database of Genomic Variants (DGV) that were >500bp and <2MB in size and did not overlap any other regions [26,30]. In total, the arrays interrogate 20,092 highly confident and distinct CNV regions in a single assay, with each CNV region surveyed by 6-9 probes. The raw signal intensities of each probe were normalized across the entire array to limit potential bias due to dye normalization and technical errors. Log_2_ ratios of each probe were calculated using the normalized intensities of the Cy5 (sample) and Cy3 (reference) channels. We then assessed all probes for variability using the Bioconductor package CNVTools, and eliminated probes without variability. A mean Log_2_ ratio for each CNV region was then calculated, and is directly analyzed (total N after QC = 17,957 autosomal CNV regions). CNV frequency calls were based on CNVTools, with the largest bin assumed to be the 2-copy version. For validation, a small subset of regions were genotyped for copy number by real-time PCR with the Applied Biosystems Taqman copy number assay on a 7900HT instrument [31], which gives continuous copy number values. The Institutional Review Boards of the Brigham and Women’s Hospital and of the other CAMP study centers approved this study. Informed assent and consent were obtained from the study participants and their parents to collect DNA for genetic studies.

## Results

### Simulation study

To assess the performance of the family-adjustment algorithm under various scenarios, we performed a simulation study. We generated intensity data based on similar scenarios in [13]. Only copy number losses were considered. The parental genotypes (0,1, or 2 copies) were generated from the distributions under Hardy-Weinberg Equilibrium for minor allele frequency ranged from 0.1-0.3. The offspring genotypes were generated conditional on the parental genotypes as in the inheritance matrix (Additional file 1: Table S1) with fixed parameters *a* = 0.0009 and *e* = 0.01 (as in [11,21]). Gaussian noises were added for various signal-to-noise ratios. For each scenario 1,000 trios (3,000 samples) were simulated for 1,200 independent CNV regions.

**Figure 1:**
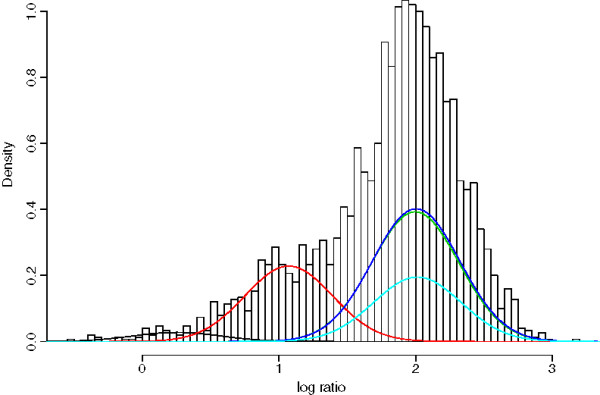
**Simulation: Gaussian mixture models.** Gaussian Mixture Model fit for one of the simulated CNV regions with MAF=0.1 and SNR=3. The Gaussian mixture components are shown in different colors and overlaid the histogram.

**Figure 2:**
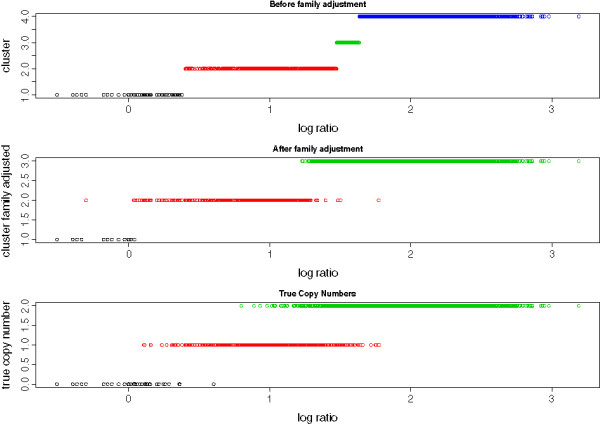
**Simulation: Before and after family adjustment.** The raw intensity values and CNV calls from the same simulated CNV regions in Figure 1. The colors denote the clusters from Gaussian mixture models (Top) and Family adjustment algorithm (Middle). The bottom panel shows the real copy numbers from which the intensity data were generated.

Table 2 shows the sensitivity, specificity and overall accuracy rate for all scenarios considered. In most cases, the two methods performed similarly in terms of overall accuracy, though the family adjustment gave slight improvement in majority of the scenarios, including all the low-noise cases (SNR ≥ 5). The family adjustment algorithm also gave more conservative CNV calls, which resulted in slightly lower sensitivities and higher specificities. The exception was the high-noise low MAF group (SNR=3 and MAF=0.1), where the family adjustment showed significant improvement. In this nosier situation, which is observed often in real data sets, the GMM sometimes gave extra clusters and the family adjustment can collapse them down to the correct number of clusters. For example, Figure 1 shows an example where the GMM chose 5 clusters as the one with highest likelihood (one of which did not have any sample assigned to it), and after family adjustment, the model collapsed down to 3 clusters and gave more accurate CNV calls (See Figure 2).

**Table 2:** Sensitivity and specificity before and after family adjustment in simulation study

	**Sensitivity**					
	**SNR**	**3**	**4**	**5**	**6**	**7**
MAF=0.1	Unadjusted	0.9095	0.9240	0.9773	0.9942	0.9988
	Family adjusted	0.7106	0.9114	0.9757	0.9937	0.9987
MAF=0.2	Unadjusted	0.9340	0.9777	0.9946	0.9990	0.9991
	Family adjusted	0.8828	0.9698	0.9922	0.9984	0.9997
MAF=0.3	Unadjusted	0.9368	0.9796	0.9925	0.9852	0.9812
	Family adjusted	0.8570	0.9733	0.9950	0.9990	0.9991
	**Specificity**					
	**SNR**	**3**	**4**	**5**	**6**	**7**
MAF=0.1	Unadjusted	0.2867	0.9789	0.9946	0.9990	0.9999
	Family adjusted	0.9411	0.9864	0.9961	0.9993	0.9999
MAF=0.2	Unadjusted	0.8975	0.9591	0.9776	0.8582	0.6295
	Family adjusted	0.9468	0.9740	0.9917	0.9981	0.9997
MAF=0.3	Unadjusted	0.9353	0.9800	0.9919	0.9812	0.9760
	Family adjusted	0.8991	0.9684	0.9927	0.9984	0.9990
	**Overall accuracy**					
	**SNR**	**3**	**4**	**5**	**6**	**7**
MAF=0.1	Unadjusted	0.5253	0.9707	0.9838	0.9878	0.9888
	Family adjusted	0.9274	0.9697	0.9838	0.9878	0.9888
MAF=0.2	Unadjusted	0.9244	0.9704	0.9620	0.8996	0.7848
	Family adjusted	0.9152	0.9708	0.9915	0.9980	0.9997
MAF=0.3	Unadjusted	0.8761	0.9709	0.9895	0.9809	0.9761
	Family adjusted	0.8282	0.9545	0.9896	0.9977	0.9987

### Application on real data

For the real data application, we refitted the Gaussian mixture model to an aCGH dataset of a genome-wide CNV association study of asthma. 14,234 polymorphic (i.e. those with 2 or more clusters) CNV regions assayed on the custom-designed array were evaluated. The GMM was applied with same fixed parameters *a* and *e* as in the simulation study for the weighted E-M algorithm. Family adjustment markedly reduced the number of copy number gains (3-4 copies) and losses (0-1 copies) observed across the cohort: when considering all loci, the total number of gains and losses was reduced by 55.3%, decreasing from 5,385,285 (31.24% of all samples/regions) to 2,409,632 (14.15%). Despite this very substantial drop in copy number variability, the overwhelming majority of markers remained polymorphic - only 177 of 14,234 (1.2%) were reclassified as monomorphic – confirming that the primary effect of family-based adjustment is the reclassification of individual alleles while retaining polymorphic distributions, rather than simply constricting population variability. This point is emphasized when analysis was restricted to the subset of loci with of common CNV (>5% frequency) that clustered discretely with high confidence (80% of the samples with calls of at least 99% posterior probability at the final E-step). Among 1,319 regions fulfilling these stringent criteria, family adjustment reduced the number of observed alleles by only 3.2% (compared to 55.3% among all regions). Thus, our method appears to operate appropriately, weeding out large proportions of alleles in questionable regions, while making much more subtle changes to high-confidence CNVs.

**Figure 3:**
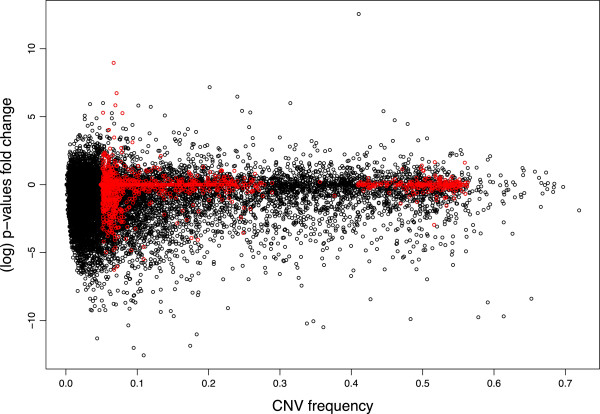
**The p-values shift after family adjustment.** The log fold changes of p-values for association testing after family adjustment for all 14,234 regions (black) and 1,319 “high confidence” regions. CNV frequency is defined as the percentage of subjects in our population with copy number gain or loss.

**Figure 4:**
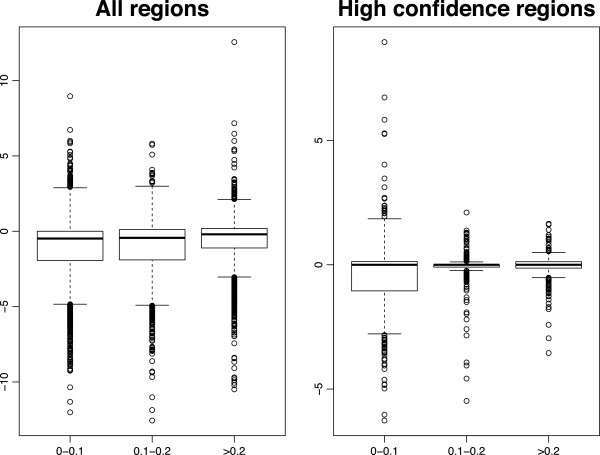
**The p-values shift after family adjustment.** Boxplots of log p-values fold changes by CNV frequency.

**Figure 5:**
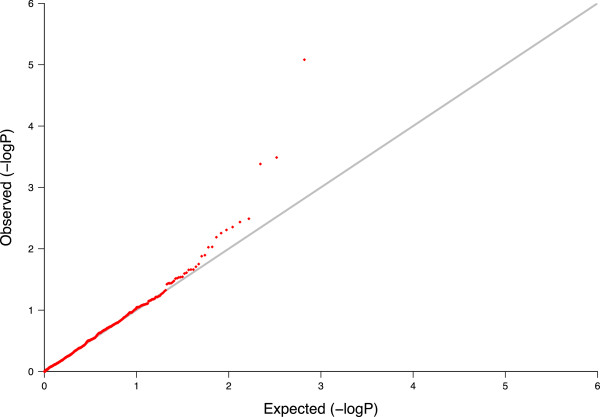
**QQ-plot for asthma association test after family adjustment.** The QQ-plots after family adjustment for 661 “high confidence” regions with CNV frequency greater than 10%.

We next assessed the impact of family-based adjustment on association testing. Using the genome-wide aCGH data in 385 parent-child trios, we applied the CNV-FBAT algorithm [18] both before and after family adjustment. Given that the adjustment procedure used local family data which aims to reconcile differences between parental and offspring copy number abundance, and because the association test assesses for differences between the observed offspring copy number and that expected from parental data, there was concern about the method possibly introducing systematic null bias and reducing statistical power. We therefore examined the effects of family-based adjustment on the distribution of association p-values for the 1,319 “high confidence” CNV regions. If bias were introduced, we would expect to observe a general asymmetry in direction of change in the magnitude of association p-values, with larger (less significant) association p-values observed post-adjustment. We found no evidence of such an effect: though 357 regions (27%) demonstrated increased (less significant) p-values following adjustment, 538 regions (40.1%) had decreased (more significant) p-values after family adjustment, and 424 (32%) remained unchanged. Using an arbitrary p-value of 0.05 cut-off, 60 CNV regions demonstrated association with asthma prior to family-based adjustment, while 104 regions were found with significant association after adjustment. Of these, 41 regions were significant both before and after adjustment. Figures 3 and 4 show the p-value fold changes for regions with different CNV frequency, and we can see that for the 1,319 high confidence regions (shown in red) the majority of regions with significant change in p-values were the relatively rare ones (CNV frequency 5-10%). The instability of association testing in rare CNVs resulted from the reduced power of the association testing resulting at small sample sizes (i.e. small number of informative families) for these rare regions, and did not suggest that the family-based adjustment itself gave less accuracy at lower allele frequencies. For the regions with CNV frequency >10*%* the results appeared to be more stable and the QQ plot shows that the family adjustment did not introduce any systematic bias in the association tests in either direction (see Figure 5).

We also assessed the utility of our method in the analysis of rare variants. We focused on 50 CNV regions overlapped or near known asthma candidate genes [32-34] with frequency ≤5*%*. After adjustment for family information, the total number of CNV went down 35% (down 48% for the offsprings, see Table 3). In particular, the total number of de novo CNVs dropped from 227 to 73 (down 68%). Though this adjusted de novo rate was higher than that expect, our algorithm eliminated the a substantial proportion of de novo CNV calls, reducing their prevalence to a more reasonable model of the true prevalence of de novo CNV.

**Table 3:** Rare CNVs in 50 regions overlapped or near known asthma genes

	**Total CNV**	**Offsprings CNV**	**De novo**
Gaussian mixture model	1157	398	227
Family-adjusted	749	205	73

**Figure 6:**
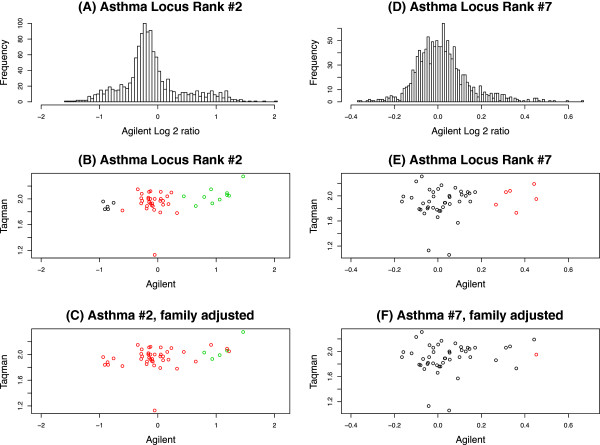
**Histograms and scatter plots for 2 asthma-associated CNV regions validated with qPCR.** The histograms (panels **A/D**) are of all samples in the two asthma CNV regions. The scatter plots (panels **B/C/E/F**) are of the 46 samples with both Agilent array and qPCR measurements. (x-axis) represents the log2 ratios from CGH arrays and the y-axis represents the copy number estimates from qPCR. The scatter plots for unadjusted GMM (panels **B/E**) and family adjusted (panels **C/F**) are the same but colored differently indicating CNV calls (clusters).

Figure 6 provides an illustration of the effects of family-based adjustment at two loci that initially demonstrated strong association with asthma pre-adjustment, but dropped out (were no longer significant) post-adjustment: Asthma Locus Rank #2 (*p*=0.0013 pre-adjustment; *p*=0.2635 post-adjustment), and Asthma Locus Rank #7 (*p*=0.0126 pre-adjustment; *p*=0.7055 post-adjustment). Despite both markers demonstrating distributions consistent with variable copy number (panels A and D) that formed fairly discrete clusters, these GGM-derived clusters were largely inconsistent with Mendelian inheritance (Panels B and E). Following family-based adjustment, many of the questionable calls were dropped, substantially reducing the number of parent-child genotype inconsistencies (Panels C and F). Indeed, independent technical validation of both markers in a subset of subjects by quantitative PCR (qPCR) confirmed that neither region is likely truly copy-number variable, further demonstrating the utility of family-based normalization processes in reducing false positive results.

**Figure 7:**
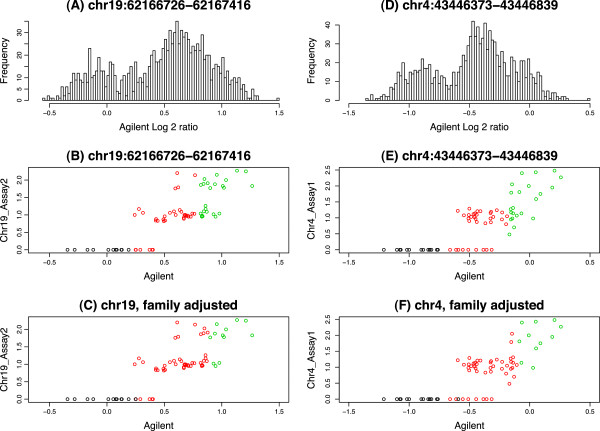
**Histograms and scatter plots for 2 CNV regions validated with qPCR.** The histograms (panels **A/D**) are of all samples in the two CNV regions. The scatter plots (panels **B/C/E/F**) are of the 72 samples with both Agilent array and qPCR measurements. Agilent (x-axis) represents the log2 ratios from CGH arrays and the y-axis represents the copy number estimates from qPCR. The scatter plots for unadjusted GMM (panels **B/E**) and family adjusted (panels **C/F**) are the same but colored differently indicating CNV calls (clusters).

Although we can see that the family adjustment algorithm generally reduce the number of CNV calls and false-positives, it is important to know how the algorithm performs when the CNVs are real. To demonstrate this point, we performed qPCR on four CNV regions with frequency ≥5*%* after family adjustment, where over 5% of the samples were reassigned (Figure 7). Even though these loci were selected based on the appearance of their array based data, as observed in other datasets, we noticed that our array-based data was noisier and not as well-clustered, as compared to that generated by qPCR. Using qPCR as gold-standard, we found that with family adjustment, the overall accuracy of CNV calling, and the correlation between array-based and qPCR copy number calls slightly improved (Figure 7 and Table 4), suggesting that family adjustment did not harm (and seemed to marginally improve) calling, even for high-confidence CNV regions.

**Table 4:** Confusion matrix for copy number estimates using qPCR and GMM from Agilent CGH arrays

		**Agilent CGH arrays GMM results** **Region 1 (chr19:62166726-62167416)**			
			**2**	**3**	**4**
	2	Unadjusted	11	6	0
		Family adjusted	(12)	(5)	(0)
	3	Unadjusted	0	26	11
		Family adjusted	(0)	(34)	(3)
qPCR results	4	Unadjusted	0	4	13
		Family adjusted	(0)	(8)	(9)
		**Region 2 (chr4:43446373-43446839)**			
			**0**	**1**	**2**
	0	Unadjusted	14	9	1
		Family adjusted	(15)	(9)	0
	1	Unadjusted	0	26	10
		Family adjusted	(0)	(34)	(2)
qPCR results	2	Unadjusted	0	0	11
		Family adjusted	(0)	(2)	(9)

The numbers in parenthesis show the estimates after family adjustments. The qPCR estimates are rounded off the nearest integer and shifted to correspond to the CGH array estimates, which designate the cluster closest to zero as the two copy group. The overall accuracy goes from 70% to 77% for region 1 (chr19:62166726-62167416) and from 72% to 82% for region 2 (chr4:43446373-43446839).

## Discussion

We have introduced a formal statistical framework to CNVs in family-based designs, using Gaussian mixture models. This method considers both the family relationships and the log2 ratios for each individual, therefore reducing the number of Mendelian inconsistencies while allowing the detection of de novo events. Results from analysis of CAMP CNV data shows that our method improves CNV calls accuracy and reduces the number of Mendelian errors and false positive CNV calls, for both common and rare CNV regions and the results can be validated with qPCR. Though we only included parent-child trios in our study, the method can easily be extended to larger pedigrees with multiple generations of families. Our method works especially well for regions with moderate data quality, as opposed to extremely well-clustered or poor data. For well-clustered regions, the Gaussian mixture models give extremely high confidence (close to 100% posterior probability) for CNV calls, therefore the re-weighting with family data will not change the results by much. On the other hand, a poorly-clustered region often contains many mendelian-incompatible trios that the algorithm cannot reconcile. Therefore, our method is most useful for the “questionable” regions where the family data can help identify the real CNV regions.

We also examined the effects of family-based adjustment on association testing. Though it is possible to perform CNV association testing using either raw intensity data or derived copy number, others and we note the later is more preferable in most situations [18], motivating the need for reliable CNV copy number calling algorithms. Since our algorithm reduces the number Mendelian errors and the number of CNV calls in general, one potential concern is that our method may have removed some real de novo events and introduced bias in the downstream association tests. Even though we may not know if the de novo events are really false positive, previous studies have suggested that de novo CNV mutation is likely rare (about 1% in healthy controls) [35-37]. We use a prior de novo rate *e*=0.01, which is close to the estimated de novo rate of 0.012 from an asthma study [37]. Even though the study focused on large CNV region (>100 Kb) and the real de novo rate in our study may be higher, from our CGH data we still observed de novo mutation rate well above previously estimated (57% before family adjustment, 36% after, see Table 3), including those estimated using high-resolution arrays and including small CNVs. Since the prior de novo rate is small, it would require stronger evidence to claim true de novo events. Therefore, the reduced de novo events after family adjustments suggest the algorithm appropriately reduced the number of false positive “de novo” events.

Compared to other current methods for family-based CNV studies, such as PennCNV [11,21], our method is more suitable for CGH arrays, where allele frequency information is unavailable. Our method is also designed for CNV association arrays, rather than CNV discovery arrays, as we do not consider the spatial correlations between adjacent probes like in the HMM methods. Our method models the family inheritance based on most of the same assumptions in [21], however, by considering each region independently, our method is much less computationally intensive, and the implementation is simply a matter of calling R functions in the existing R packages mclust and cnvtools. Finally, we note that our methodology is not influenced by the manner in which CNV regions are defined, as it can be applied on probe level data as well.

## Conclusions

In conclusion, though our method does not completely solve the data quality issue for CNV studies, we have shown through our analysis that incorporation of family data is a necessary step for better quality CNV calls which hopefully lead to more powerful family-based CNV association tests.

## Competing interests

The authors declare that they have no competing interests.

## Authors’ contributions

JC developed the main mathematical models and implemented the algorithm. Additional analyses were performed by AR and IIL. KD, RM and CL designed the CGH array and performed the assay for CNV association study for asthma. BAR is principal investigator of the primary grant supporting this work, “Structural Genetic Variation in Asthma” and together with JC conceptualized the algorithm. JC and BAR were responsible for manuscript preparation. All authors have read the manuscript and approved the final version.

## Supplementary Material

Additional file 1 **Table S1.** Conditional probability table. The Conditional probability of total copy number of an offspring (O) given the copy number of mother (M) and father (F). The parameter *e* denotes the probability of de novo events and *a* denotes the probability of the rare chromosome-specific copy number configuration. Click here for file
