# Supplementary material for: Copy number variation genotyping using family information
Source: BMC Bioinformatics. 2013 May 9;14:157. doi: 10.1186/1471-2105-14-157 (PMC3668900; doi:10.1186/1471-2105-14-157)
Supplement: Additional file 1 — Table S1. Conditional probability table. The Conditional probability of total copy number of an offspring (O) given the copy number of mother (M) and father (F). The parameter e denotes the probability of de novo events and a denotes the probability of the rare chromosome-specific copy number configuration. [file 1471-2105-14-157-S1.pdf]

		F0	F1	F2	F3	F4
M0	O0	1-e				
	O1	0.25e				
	O2	0.25e				
	O3	0.25e				
	O4	0.25e				
M1	O0	0.5(1-e)	0.25(1-e)			
	O1	0.5(1-e)	0.5(1-e)			
	O2	0.333e	0.25(1-e)			
	O3	0.333e	0.5e			
	O4	0.333e	0.5e			
M2	O0	0.5a(1-e)+0.25e	0.25a(1-e)+0.3333e	$0.25a^2(1-e)+0.25e$		
	O1	(1-a)(1-e)	(0.5-0.25a)(1-e)	a(1-a)(1-e)+0.25e		
	O2	0.5a(1-e)+0.25e	(0.5-0.25a)(1-e)	$((1-a)^2+0.5a^2)(1-e)$		
	O3	0.25e	0.25a(1-e)+0.3333e	a(1-a)(1-e)+0.25e		
	O4	0.25e	0.3333e	$0.25a^2(1-e)+0.25e$		
M3	O0	0.5a(1-e)+0.3333e	0.25a(1-e)+0.5e	$0.25a^2(1-e)+0.3333e$	$0.25a^2(1-e)+0.5e$	
	O1	0.5(1-a)(1-e)	0.25(1-e)	0.75a(1-a)(1-e)+0.3333e	0.5a(1-a)(1-e)+0.5e	
	O2	0.5(1-a)(1-e)	0.5(1-a)(1-e)	$(0.5(1-a)^2+0.25a(1-a)+0.25a^2)(1-e)$	$(0.5a(1-a)+0.25(1-a)^2)(1-e)$	
	O3	0.5a(1-e)+0.3333e	0.25(1-e)	$(0.5(1-a)^2+0.25a(1-a)+0.25a^2)(1-e)$	$(0.5(1-a)^2+0.5a^2)(1-e)$	
	O4	0.3333e	0.25a(1-e)+0.5e	$(0.75a(1-a)+0.25a^2)(1-e)+0.3333e$	$(0.25(1-a)^2+a(1-a)+0.25a^2)(1-e)$	
M4	O0	0.5e	e	0.5e	0.5e	0.5e
	O1	0.25(1-e)	0.125(1-e)	0.125a(1-e)+0.5e	0.125a(1-e)+0.5e	0.5e
	O2	0.5(1-e)	0.375(1-e)	0.25(1-e)	0.125(1+a)(1-e)	0.0625(1-e)
	O3	0.25(1-e)	0.375(1-e)	(0.5-0.25a)(1-e)	0.125(3-2a)(1-e)	0.25(1-e)
	O4	0.5e	0.125(1-e)	(0.25+0.125a)(1-e)	0.5(1-e)	0.6875(1-e)

The Conditional probability of total copy number of an offspring(O) given the copy number of mother(M) and father(F). The parameter **e** denotes the probability of de novo events and **a** denotes the probability of the rare chromosome-specific copy number configuration.
